# The Determining Influence of the Phase Composition on the Mechanical Properties of Titanium—Iron Alloys after High-Pressure Torsion

**DOI:** 10.3390/ma17153740

**Published:** 2024-07-28

**Authors:** Alena S. Gornakova, Boris B. Straumal, Alexander I. Tyurin, Natalia S. Afonikova, Askar R. Kilmametov, Alexander V. Druzhinin, Aleksey N. Nekrasov, Gregory S. Davdian, Luong V. Duong

**Affiliations:** 1 Osipyan Institute of Solid State Physics of the Russian Academy of Sciences, Ac. Osipyan str. 2, Chernogolovka 142432, Russia; straumal@issp.ac.ru (B.B.S.); natasha@issp.ac.ru (N.S.A.); druzhinin@issp.ac.ru (A.V.D.); faberest@yandex.ru (G.S.D.); 2 G.R. Derzhavin Research Institute “Nanotechnologies and Nanomaterials” TSU, Internazionalnaja str. 30, Tambov 392000, Russia; tyurin@tsu.tmb.ru; 3 Laboratory of Technological and Materials Science Research, 93430 Viltanez, France; askar.kilmametov@univ-paris13.fr; 4 Korzhinskii Institute of Experimental Mineralogy, Russian Academy of Sciences, Ac. Osipyan str. 4, Chernogolovka 142432, Russia; alex@iem.ac.ru; 5 Institute of Materials Science, Vietnam Academy of Science and Technology, 18 Hoang Quoc Viet Road, Cau Giay District, Hanoi 70072, Vietnam; duonglv@ims.vast.ac.vn

**Keywords:** titanium alloys, pre-annealing, high-pressure torsion, phase transitions, nanoindentation

## Abstract

Three titanium alloys with 0.5, 6, and 9 wt.% iron were investigated, and the samples were pre-annealed in three different regions of the Ti–Fe phase diagram, namely β, α+β, and α+FeTi. After annealing, five samples of different phases and structural compositions were studied. They were then subjected to the high-pressure torsion (HPT). The microstructure of the samples before and after HPT treatment was studied using transmission and scanning electron microscopy. The microstructure of the samples obtained during heat treatment before HPT treatment had a fundamental effect on the microstructure after HPT. Grain boundary layers and chains of particles formed during the annealing process made it difficult to mix the material during HPT, which led to the formation of areas with non-uniform mixing of components. Thus, the grain boundary layers of the α-phase formed in the Ti–6wt % Fe alloy after annealing at 670 °C significantly decreased the mixing of the components during HPT. Despite the fact that the microstructure and phase composition of Ti–6wt % Fe alloys pre-annealed in three different regions of the Ti–Fe phase diagram had significant differences, after HPT treatment, the phase compositions of the studied samples were quite similar. Moreover, the measured micro- and nanohardness as well as the Young’s modulus of Ti–6wt % Fe alloy had similar values. It was shown that the microhardness of the studied samples increased with the iron content. The values of nanohardness and Young’s modulus correlated well with the fractions of β- and ω-phases in the studied alloys.

## 1. Introduction

Since R.Z. Valiev and his co-authors described a method for severe plastic deformation (SPD) [1,2], the interest in materials subjected to SPD has not subsided to this day. SPD allows not only for the reduction of the grain size of bulk metal samples down to the nanoscale but also a change in the phase composition of the material as well as its physical and mechanical properties. Fundamental parameters of materials are typically changed upon SPD, such as elastic modules, saturation magnetization, etc. This, in turn, opens up ways for improving existing materials and creating fundamentally new ones. Additionally, the phase composition, microstructure, and mechanical properties of alloys after SPD are affected by applied pressure [3], temperature during deformation [4], impurities [5,6], the number of anvil revolutions [5,7], the anvil rotation speed [8], as well as grain orientation in the starting material [7]. In our work, we exclude most of the above-mentioned factors by fixing the experimental parameters.

In our work, titanium alloys subjected to high-pressure torsion (HPT) were studied. The HPT of pure titanium has been studied in numerous studies [9,10,11,12,13,14]. Similarly, there are numerous published studies in the literature devoted to the HPT of binary titanium alloys like Ti–Al [15,16,17,18,19,20,21,22,23,24], Ti–Mg [25,26], Ti–Fe [27], Ti–Ni [28,29,30,31,32,33,34,35,36,37,38,39,40,41,42,43,44,45], Ti–Mo [46,47,48,49,50,51,52,53,54], Ti–Nb [50,51,55,56,57,58,59,60,61,62,63,64,65,66], Ti–Hf [67], Ti–V [68], and Ti–Ta [50]. HPT is one of the SPD modes that allows for obtaining the finest grains. To investigate mechanical properties, nanoindentation was chosen [69]. Nanoindentation allows for measuring almost non-destructively the nanohardness and calculating Young’s modulus. Modern nanoindentation makes it possible to study mechanical properties of various materials at the nanoscale. It can also complement the complex standard methods, providing the possibility to re-examine samples.

Recently, the Ti–Fe binary alloys have attracted considerable attention in the automotive and aerospace industries. First of all, this is due to their lower price ensured by the absence of expensive alloying elements, such as vanadium, molybdenum, and niobium. Another reason is the wide range of microstructures and mechanical properties obtained in Ti–Fe alloys [70,71,72]. Basically, the microstructures of titanium alloys can consist of two main phases, namely the β-phase with a body-centered cubic lattice (BCC), which is stable at high temperatures, and the low-temperature α-phase with a hexagonal close packed lattice (HCP). However, when titanium and its alloys are subjected to HPT, an additional high-pressure phase (ω-phase) is typically detected. It appears under shear strain under pressure, and it is conserved after pressure release and even after additional heating up to 300–500 °C.

Earlier, the present authors and their colleagues thoroughly studied titanium–iron alloys [73,74,75,76,77,78,79,80] with a different fraction of the second component and various kinds of heat treatments, also subjected to HPT, as one of the most popular SPD modes. One of the main conclusions of these studies is that the second component and preliminary heat treatment determine the phase composition in the samples subjected to HPT, particularly the fraction of the ω-phase. The purpose of this work is to find the relationship between the proportion of alpha/beta/omega phases and nanohardness/Young’s modulus in samples subjected to the thermal and HPT treatments.

## 2. Materials and Methods

**Figure 1:**
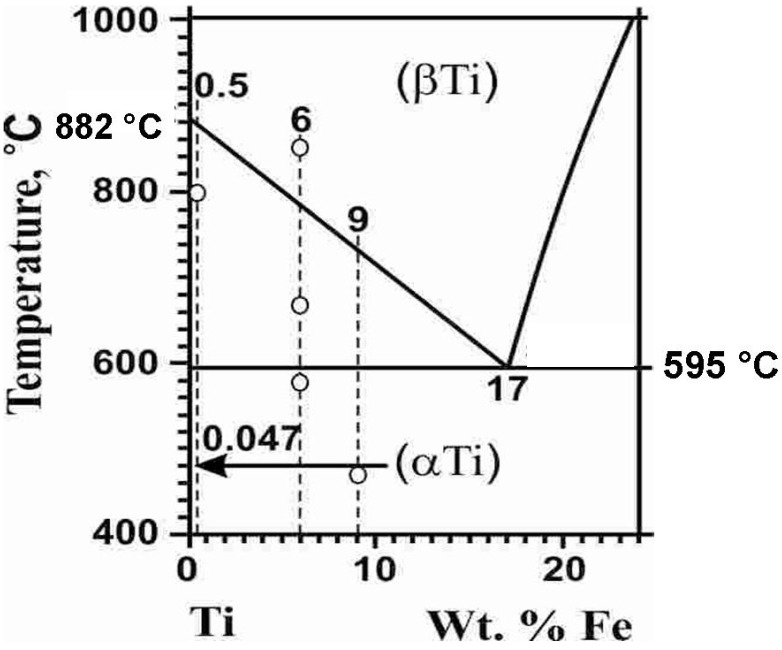
The part of the Ti–Fe phase diagram with marked annealing temperatures (circles).

The investigated binary titanium alloys, Ti–0.5 wt.% Fe (0.5 ± 0.0 wt.% Fe), Ti–6 wt.% Fe (6.3 ± 0.3 wt.% Fe), and Ti–9 wt.% Fe (9.2 ± 0.2 wt.% Fe), were manufactured in the form of cylindrical ingots with a diameter of 2*R* = 10 mm. The levitation melting method in an atmosphere of pure argon using electromagnetic induction was used for this purpose. These alloys were smelted using pure titanium (99.98%, TI-1 grade, Special Metallurgy Ltd., Moscow, Russia) and iron (99.97%, Special Metallurgy Ltd., Moscow, Russia). Chemical analysis of ingots was carried out with the help of a scanning electron microscope Tescan Vega TS 5130 MM (Brno, Czech Republic), equipped with an energy-dispersion spectrometer LINK (Oxford Instruments, Abingdon, UK). SEM images were made using Versa 3D DualBeam FEI (Thermo Fisher Scientific, Hillsborough, OR, USA). The 0.7 mm thick disks were cut off from the ingot using spark erosion. After removing the disturbed surface layer by grinding, the samples were annealed in the sealed quartz ampoules with the initial residual pressure in the ampoule of about 4 × 10^−4^ Pa. Annealing was carried out in various regions of the titanium–iron phase diagram (Figure 1): 800 °C, 7 h (Ti–0.5 wt.% Fe), 580 °C, 840 h, 670 °C, 1230 h, 850 °C, 148 h (Ti–6 wt.% Fe), and 470 °C, 673 h (Ti–9 wt.% Fe). The samples were quenched after annealing in cold water together with the ampoule.

Afterwards, the annealed and quenched samples were subjected to HPT. The HPT conditions were room temperature with 5 rotations of the plunger, under pressure of 7 GPa, at a rotation speed of 1 rpm. HPT was performed in a computer-controlled Bridgman anvil-type machine manufactured by W. Klement GmbH, Lang, Austria. After the HPT, the thickness of the samples was 0.35 mm. The nanohardness of the samples was measured using the Hysitron TI-950 Triboindenter (Bruker, Eden Prairie, MN, USA) equipped with a Berkovich indenter. Measurements were carried out along the radius of the samples, and the loading rate was constant and equal to d*P*/d*t* = 40 mN/s. Before the measurements, the surface of the samples was polished using a diamond paste with a grain size of 1 µm. The numerical values of nanohardness (*H*) and Young’s modulus (*E*) of the studied samples were determined using the Oliver–Farr method based on characteristic *P-h* diagrams [81,82].

The structural and phase analysis was performed with the Rigaku Smartlab X-ray diffractometer (Rigaku Corp., Akishima-shi, Tokyo, Japan). The CuK_α1+α2_ radiation was used. The X-ray wavelength was 0.15419 nm. The phase analysis and calculation of the lattice parameters were carried out using the PowderCell 2.4 program (PowderCell for Windows. Version 2.4. 08.03.2000, Werner Kraus & Gert Nolze, BAM Berlin). After HPT, the microstructure of the samples was studied using a 200 kV transmission electron microscope JEM-2100 (TEM) (JEOL Ltd., Akishima, Japan). Samples for TEM studies were prepared using standard techniques, including mechanical polishing and etching with Ar ions.

## 3. Experimental Results

### 3.1. Microstructures and Phase Composition of Samples before and after HPT

**Figure 2:**
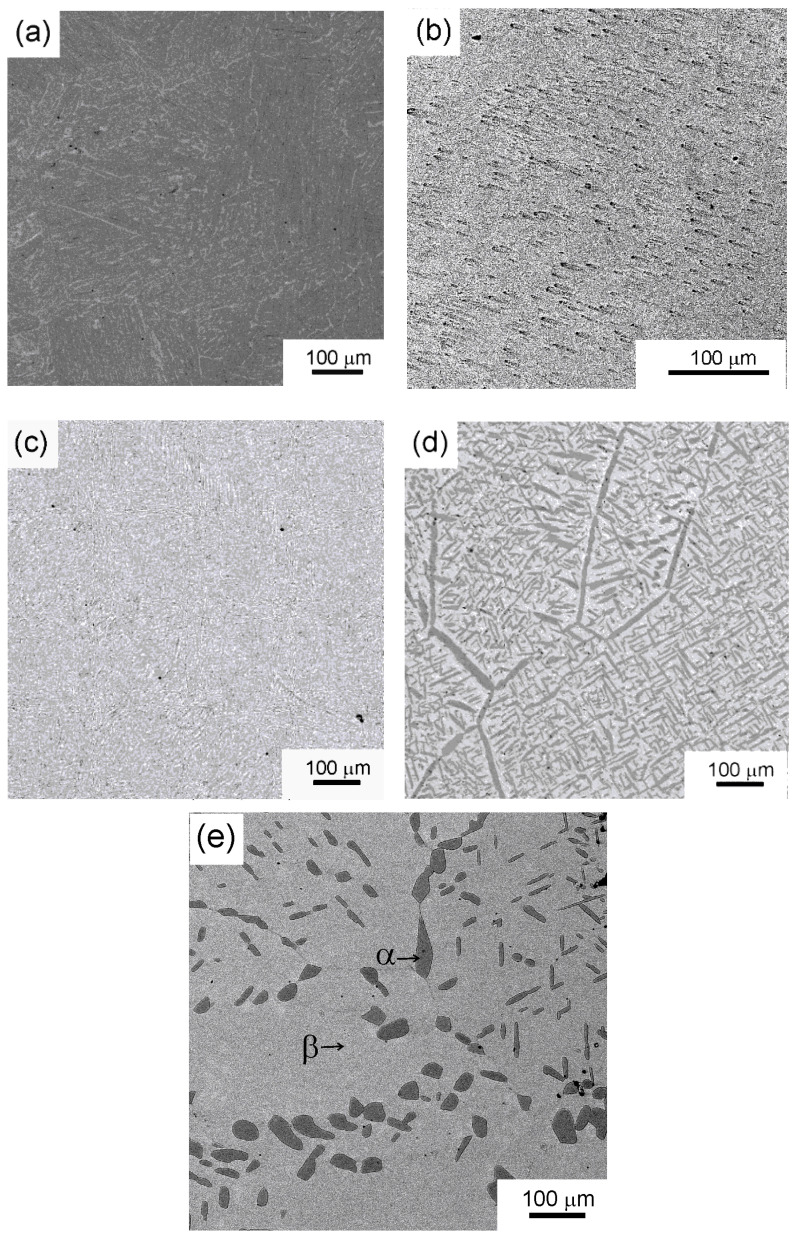
SEM images: (**a**) Ti–0.5 wt.% Fe annealed at 800 °C, (**b**) Ti–9 wt.% Fe at 470 °C, and Ti–6 wt.% Fe at (**c**) 580 °C, (**d**) 670 °C, (**e**) 850 °C.

Figure 2 shows micrographs of titanium–iron alloys annealed at various temperatures (Figure 2a), Ti–0.5 wt.% Fe, 800 °C, (Figure 2b) Ti–9 wt.% Fe, 470°C, and Ti–6 wt.% Fe (Figure 2c), 580 °C, (Figure 2d) 670 °C, and (Figure 2e) 850 °C.

**Figure 3:**
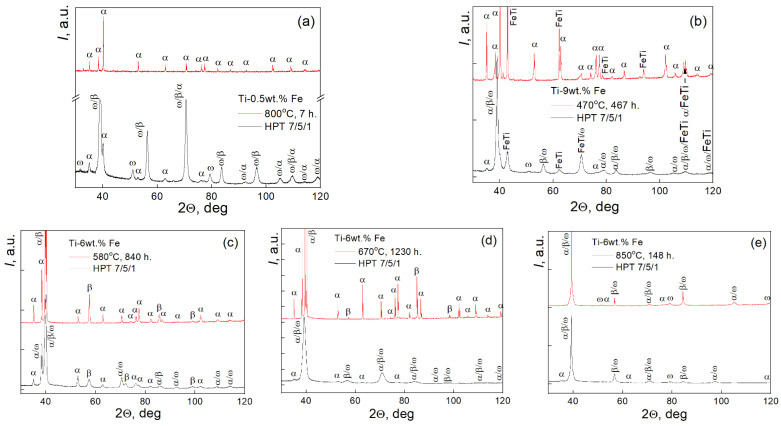
XRD patterns of samples before (red lines) and after HPT (black lines): (**a**) Ti–0.5 wt.% Fe, (**b**) Ti–9 wt.% Fe и Ti–6 wt.% Fe пpи, (**c**) 580 °C, (**d**) 670 °C, (**e**) 850 °C. “HPT 7/5/1” means HPT at 7 GPa, 5 rot., 1 rpm.

The SEM images show that the composition of the alloys and the annealing temperature have a significant impact on the microstructure before the HPT. The image in Figure 2a shows a completely single-phase (αTi) material (see also XRD results in Figure 3 and Table 1). The image in Figure 2b demonstrates that the particles of the brittle intermetallic-phase FeTi had fallen apart during polishing (black dots). Although the microstructure in Figure 2c contains 50/50% of α/β-phases, the phase distribution is uniform, and it is quite difficult to estimate the grain size. In Figure 2d,e, there is a light gray and dark gray contrast related to the α- and β-phases. In Figure 2d, the α-phase is present in both the (βTi) grains and at the (βTi)/(βTi) grain boundaries. Meanwhile, in Figure 2e, the α-phase is completely distributed along the (βTi)/(βTi) grain boundaries. It is worth noting that all five samples have different phase compositions and various microstructures after annealing. In the Ti–6 wt.% Fe alloy annealed at 850 °C, the isothermal ω-phase is visible in the XRD pattern in addition to the α- and β-phases (Figure 3b).

**Figure 4:**
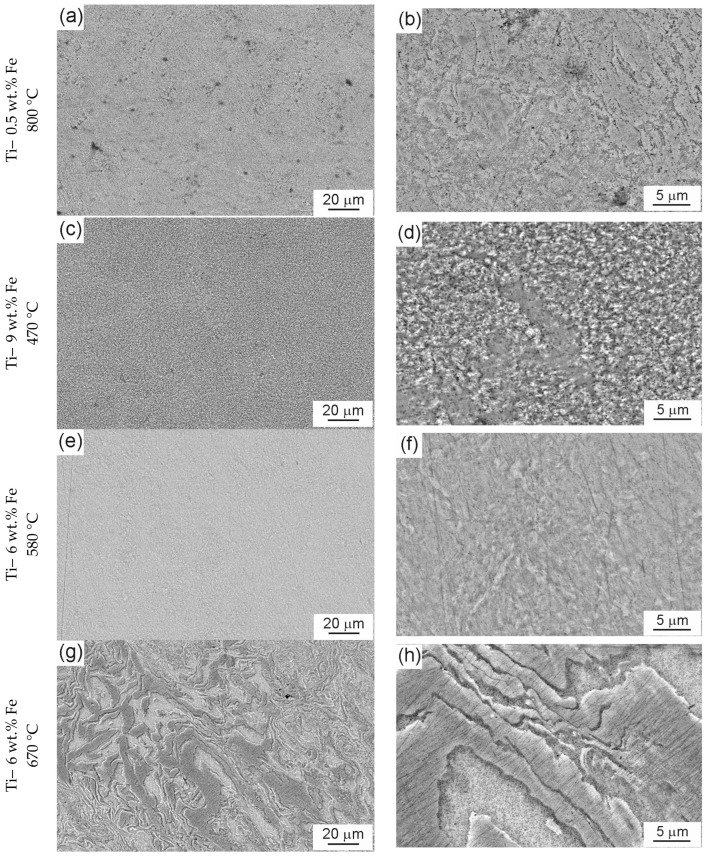

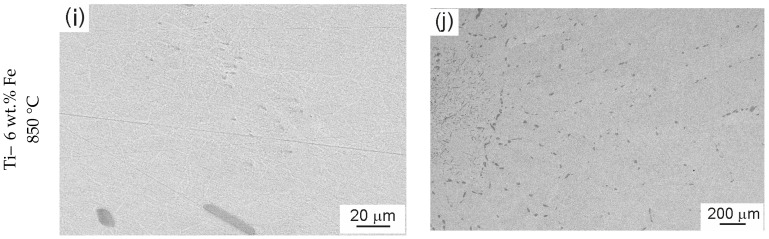
SEM micrographs HPT: (**a**,**b**) Ti–0.5 wt.% Fe, 800 °C, (**c**,**d**) Ti–9 wt.% Fe at 470 °C and Ti–6 wt.% Fe at (**e**,**f**) 580 °C, (**g**,**h**) 670 °C, and (**i**,**j**) 850 °C.

In Figure 4, the SEM micrographs of samples after HPT are presented. In Figure 4a,c,e, the distribution of iron is uniform, and the grayscale is uniform without any significant changes. In Figure 4g,i, the micrographs of two Ti–6 wt. % Fe samples pre-annealed at 670 °C (Figure 2d) and 850 °C (Figure 2e) are shown. In these samples, the GB layers of α-phase and GB chains of α-particles formed during annealing and quenching significantly decreased the mixing of the components during HPT. As a result, the regions rich and depleted of iron were formed (Figure 4h). In Figure 4j, we see that not all chains of the α-Ti phase were destroyed during HPT.

Table 2 shows the results of the X-ray diffraction analysis of samples after HPT treatment. The phase composition of the Ti–6 wt.% Fe alloy, pre-annealed at three different temperatures and having different microstructures and phase compositions, has a similar phase distribution after HPT.

**Figure 5:**
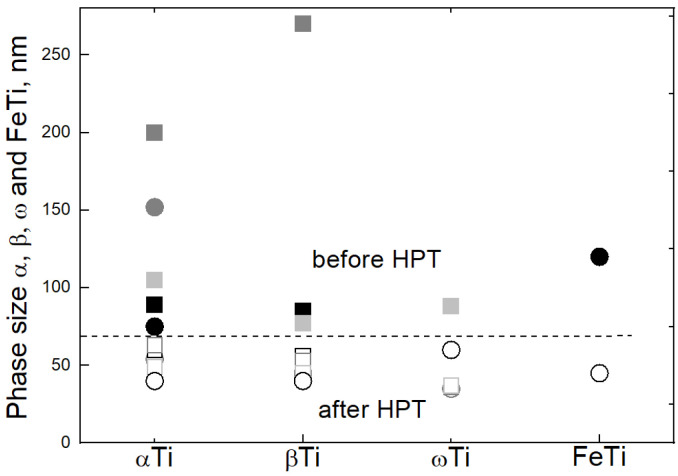
Grain size in the (αTi), (βTi), (ωTi), and FeTi phases in the annealed state (filled symbols) and after HPT (open symbols): Ti–0.5 wt.% Fe (light circles), Ti–9 wt.% Fe (dark circles), and Ti–6 wt.% Fe (squares of different colors).

The average values of the grain size in different phases before and after HPT were determined (Figure 5). These values were calculated from X-ray diffraction data. They correspond to the size of coherent scattering regions for different phases. The filled symbols correspond to the annealed samples. The data points are quite spread. The largest grains are present in the Ti–6 wt.% Fe alloy annealed at 670 °C. There are no grains smaller than 70 nm in annealed samples. HPT leads to the decrease of grain sizes, and the average grain size decreases to 50 nm. This is also evidenced by X-ray diffraction due to a characteristic broadening of peaks in HPT-treated samples. Data for the samples subjected to HPT are shown in Figure 3 and Figure 4, as well as in the Table 2. For the Ti–6 wt.% Fe alloy, the phase ratio α/β/ω varies slightly with the annealing temperature, and the mean phase ratio value is 18/36/46, respectively. A significant difference is observed in Ti–0.5 wt.% Fe and Ti–9 wt.% Fe alloys. In these alloys, the fraction of α-phase is almost equal. The fraction of ω-phase in the Ti–9 wt.% Fe alloy is two times larger (about 60%) than in the Ti–0.5 wt.% Fe alloy. The highest fraction of β-phase is in the Ti–0.5 wt.% alloy.

### 3.2. Characterization of the Microstructure of HPT-Processed Samples Using a TEM

**Figure 6:**
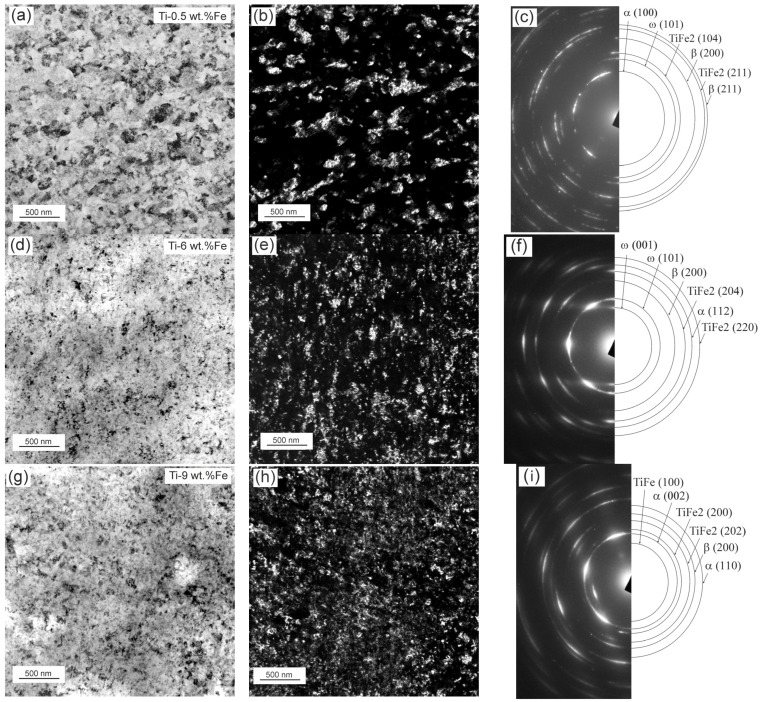
TEM images of samples after HPT: light field (**a**,**d**,**g**), dark field (**b**,**e**,**f**), and the SAED (**c**,**f**,**i**) patterns for alloys Ti–0.5 wt.% Fe (**a**,**b**,**c**), Ti–6 wt.% Fe (**d**,**e**,**f**), and Ti–9 wt.% Fe (**g**,**h**,**i**).

The microstructure of samples processed through HPT was also investigated using a TEM (Figure 6).

The electron diffraction patterns presented in the central part of Figure 6 were analyzed. The respective data are given in Table 3. The intermetallic-phase TiFe_2_ was not determined through XRD. However, it was detected using a TEM in all investigated samples. There are two assumptions about the formation of this intermetallic compound. The first is based on the emergence of the TiFe_2_ phase during the manufacture of ingots, i.e., there are non-melted Ti particles enriched with Fe. The second supposes that the intermetallic phase forms during HPT. In this paper, these are just assumptions that have no confirmation. However, another question arises: how significant is the role of these particles, and how does their volume change with an increase in the iron content in the alloy?

**Figure 7:**
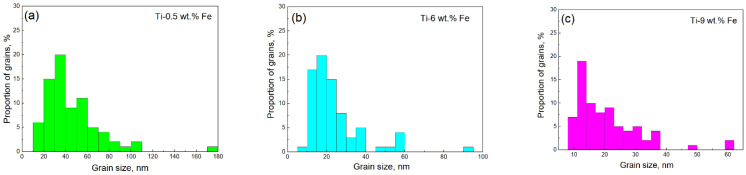
The average grain size of the phases in alloys (**a**) Ti–0.5 wt.% Fe (green color), (**b**) Ti–6 wt.% Fe (blue color), and (**c**) Ti–10 wt.% Fe (pink color) after HPT, measured in the dark field images of Figure 6.

In the dark field images (Figure 6), the average grain sizes are 35 ± 2 nm for Ti–0.5 wt.% Fe, 17 ± 1 nm for Ti–6 wt.% Fe, and 12 ± 1 nm for Ti–10 wt.% Fe (Figure 7). Because diffraction spots that formed the dark field images are unknown, it is difficult to identify the phases.

**Figure 8:**
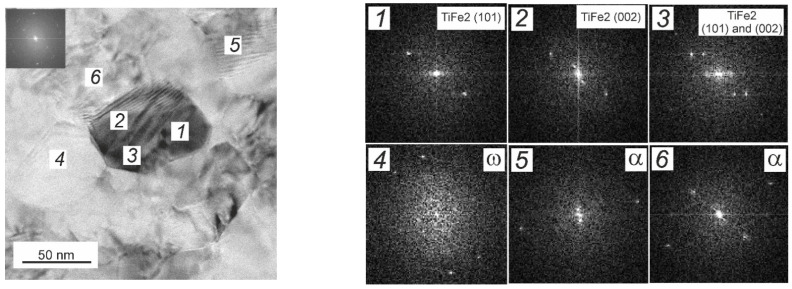
Bright field TEM image of various phases in Ti–0.5 wt.% Fe alloy after HPT. Insets 1 to 6 show FFT images in the respective points shown in the micrograph.

Confirmation of the presence of the intermetallic-phase TiFe_2_ in alloys was found using a TEM (Figure 8). The dark particle in the center of the image consists of two TiFe_2_ grains surrounded by the α and ω grains.

### 3.3. Nanoindentation and Microhardness of Samples after HPT

**Figure 9:**
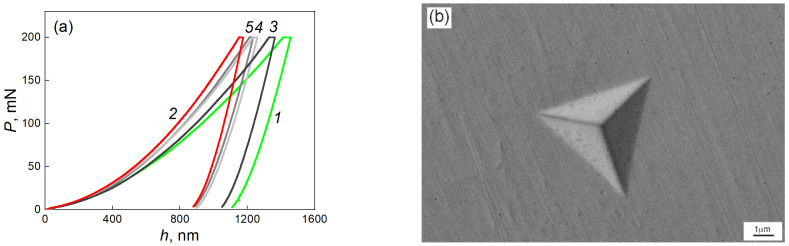
(**a**) *P*-*h* diagrams taken from the middle of the radius of samples (1) Ti–0.5 wt.% Fe, Ti–6 wt.% Fe (3) for 580 °C, (4) for 670 °C, (5) for 850 °C, and (2) for the Ti–9 wt.% Fe alloy. (**b**) SEM image of the Berkovich indenter fingerprint taken from a Ti–6 wt.% Fe sample annealed at 670 °C and subjected to HPT.

Figure 9a shows the *P-h* diagrams taken from the middle of the radius of the samples processed through HPT. The extreme curves relate to alloys with 0.5 and 9 wt.% iron. They correspond to the softest and hardest response of the material, respectively. Figure 9b shows a typical imprint of the Berkovich indenter for the studied alloys. The length of the imprint side is about 6 µm. There are no specific features on the imprint, and there are no parapets around the imprint. Based on the *P-h* diagrams, the values of nanohardness (*H*) and Young’s modulus (*E*) were calculated.

**Figure 10:**
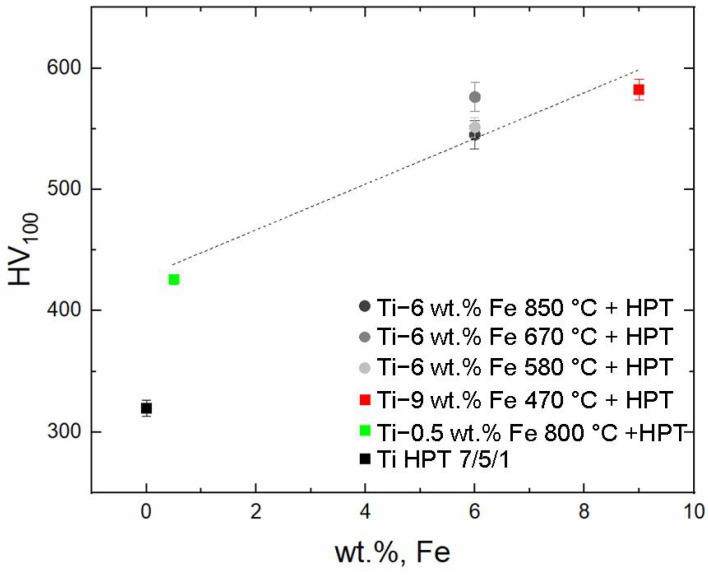
The microhardness values measured in the middle of the radius of the alloy samples Ti–0.5 wt.% Fe (green square), Ti–6 wt.% Fe (circles of various grayscale), and Ti–9 wt.% Fe (red square).

The measured values of microhardness (Figure 10) show an increase in the alloy hardness with an increase in the iron content. Alloying titanium with 0.5 wt.% Fe leads to an increase in microhardness by more than 100 HV compared with pure titanium. Alloy with 9 wt.% Fe shows a 55% increase in microhardness. It was noted that although the microstructures of the Ti–6 wt.% Fe alloy differ after HPT, the microhardness values are similar.

## 4. Discussion

**Figure 11:**
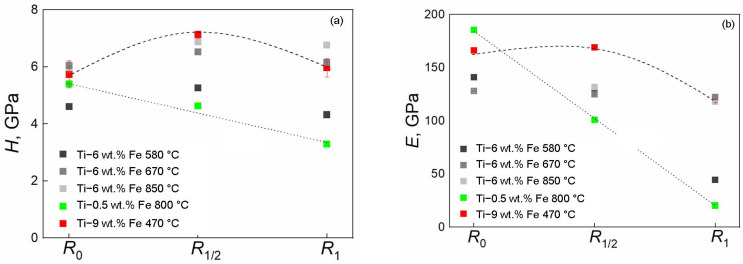
The values of nanohardness *H* (**a**) and Young’s modulus *E* (**b**) are presented for all studied Ti–Fe alloys. They were measured in three regions of the samples: in the central part (*R*_0_), in the middle of the radius (*R*_1/2_), and at the edge (*R*_1_).

Based on the data obtained from the *P-h* curves in three regions (center, middle of the radius, and edge of the sample), the values of *H* and *E* as a function of the analyzed region are shown in Figure 11. It can be seen that the values corresponding to 0.5 wt.% of iron are the smallest, but there is a linear decrease in values from the center to the edge of the HPT disc. In the center, the material has higher hardness and Young’s modulus, while the edge of the sample shows a low value of *H* = 3.3 ± 0.1 GPa and the abnormally low value of *E* = 20.4 ± 0.5 GPa. However, such “abnormal” *E* values on the edges of samples in titanium–iron alloys have been published earlier in Refs. [80,83]. The upper dotted line refers to the 9 wt.% of iron, and it has a maximum in the middle of the sample radius. The nanohardness of samples doped with 6 wt.% of Fe linearly depends on the radius, and the hardness increases. Therefore, at a pre-annealing temperature of 580 °C, the abnormally low value of *E* = 44.5 ± 0.8 GPa is again manifested at the edge of the sample.

In a Ti–Fe alloy, the iron content determines the kinetics of growth and dissolution of phases. Under high-pressure conditions, the mass transfer of iron atoms is controlled by the dislocation glide imposed by shear stresses. The high density of mobile dislocations with cores supersaturated with vacancies as well as vacancies of interphase boundaries can cause an abnormal drop in the Young’s modulus in titanium and titanium alloys [84,85]. Another reason for the decrease of the Young’s modulus at the edge of the sample in combination with the high mobility of dislocations can be the nanoporosity of the material [86]. The porosity of the material and the oxygen content in the studied alloys were not determined in this work.

**Figure 12:**
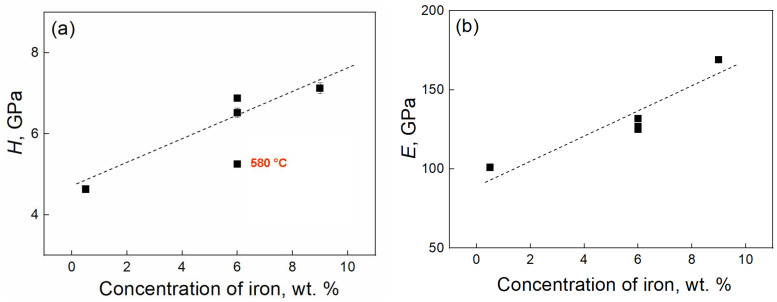
The dependences of the values of (**a**) nanohardness *H* and (**b**) Young’s modulus *E* on the iron content in Ti–Fe alloys measured in the middle of the radii of the samples.

Next, the dependences of *H* and *E* values on the iron content in Ti–Fe alloys were derived (Figure 12). The *R*_1/2_ values were taken for these plots. All dependences are linear, and the magnitudes of *H* and *E* increase with the rise of the Fe content in the alloy. There is a sharp decrease in the nanohardness value in the Ti–6 wt.% Fe alloy annealed at 580 °C, while the fraction of the ω-phase for this alloy is the highest. Based on these results, we can make an assumption based on the impact of the pre-annealing on the phase composition of samples after HPT. We can assume that the lower the pre-annealing temperature, the higher the fraction of the ω-phase in the titanium–iron alloy and the lower the nanohardness value.

**Figure 13:**
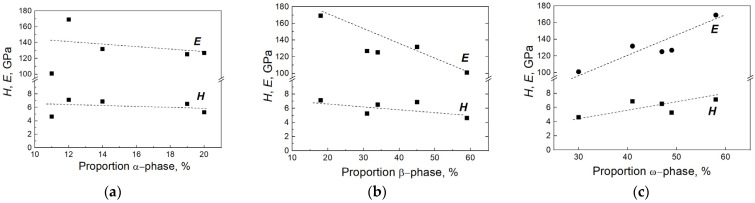
The dependences of *H* and *E* values on the fraction of α- (**a**), β- (**b**), and ω-phases (**c**) in Ti–Fe alloys.

Because the dependencies in Figure 12 are linear, we decided to check how strong the correlation among *H*, *E,* and the fractions of α-, β-, and ω-phases is based on the results shown in Figure 13. We considered all of our dependencies as linear. In the direction of correlation, it is positive/forward (Figure 13c), negative/reverse (Figure 13b), and there is no connection (Figure 13a).

In Figure 13a, the E and H values are almost independent of the portion of the α-phase and the slopes are close to zero, i.e., −0.097 and −0.028, respectively. The absence of correlation and the spread of experimental points among the values of nanohardness and Young’s modulus and the fraction of the α-phase indicates the absence of correlation of the studied parameters. For the β-phase, the E/H dependencies have correlation coefficients equal to −0.881/−0.0623 (Figure 13b), and for the ω-phase E/H they are 0.898/0.660 (Figure 13c). On the Chaddock scale for a qualitative assessment of the strength of the coupling of characteristics, the derived values are attributed to high indicators [87]. A weak spread of experimental points also indicates a high correlation of parameters. In any case, the relationship of H from the portion of β- and ω-phases is “noticeable”, and for E it is “high”.

**Figure 14:**
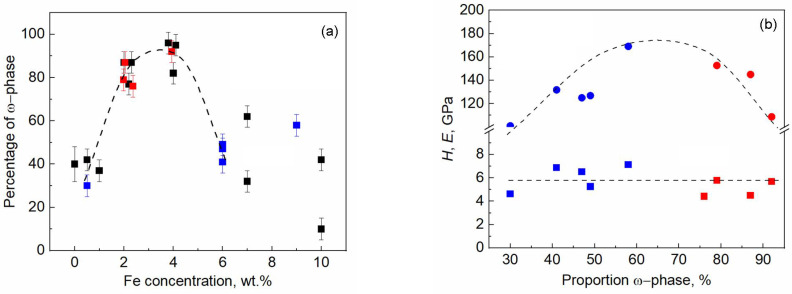
(**a**) Dependence of the fraction of the ω-phase on the concentration of iron in Ti–Fe alloys pre-annealed in various regions of the Ti–Fe phase diagram. (**b**) The dependences of *H* (squares) and *E* (circles) on the portion of the ω-phase. The blue symbols in the graphs refer to this work, the red symbols refer to Ref. [80], and the black ones refer to Ref. [74].

Combining the results of previously published studies on titanium–iron alloys [74,80] and the results obtained in this work, two graphs were constructed (see Figure 14). The blue symbols in both graphs correspond to this work. Figure 14 shows that the maximum at 4 wt% Fe remained and, until 6 wt% Fe, there is no impact of the pre-annealing before HPT. Alloying with 6 wt% of Fe results in a significant impact of the pre-annealing, and the spread of the data points is large. At the same time, the lower the pre-annealing temperature, the higher the fraction of the omega phase after HPT treatment. Figure 14b shows that the nanohardness (*H*) is around 6 ± 1.5 GPa and weakly influenced by the fraction of the omega phase, while Young’s modulus (*E*) has a maximum at 60% of the ω-phase. Additional research is needed on the mechanical properties of the Ti–4wt.% Fe alloy to confirm the dotted line shown on the dependence for Young’s modulus.

## 5. Conclusions

1. It has been shown that the main influence on the phase transformations after HPT in titanium–iron alloys is manifested by the iron content. Although the Ti–6 wt.% Fe alloy was annealed at three different temperatures and three different states of microstructures and phase compositions were obtained, nevertheless, after HPT, the phase compositions were quite similar. The nanohardness and Young’s modulus also had similar values.

2. Ti–0.5 wt.% Fe and Ti–9 wt.% Fe alloys show extreme values of nanohardness and Young’s modulus, increasing with the rise of the Fe content.

3. We would especially like to underline the high correlation among the Young’s modulus *E* and the fractions of β- and ω-phases.

## Figures and Tables

**Table 1:** Fractions of phases and parameters of phase lattices in alloys after annealing.

Fe, wt.%	*T*, °C	αTi		βTi		ωTi		FeTi	
		*V*, %	*a*, *c*, nm	*V*, %	*a*, nm	*V*, %	*a*, *c*, nm	*V*, %	*a*, nm
0.5	800	100	0.2949; 0.4684	-	-	-	-	-	-
6	580	53	0.2949; 0.4685	47	0.3206	-	-	-	-
6	670	49	0.2950; 0.4686	51	0.3220	-	-	-	-
6	850	11	0.2950; 0.4686	79	0.3243	10	0.4626; 0.2813	-	-
9	470	84	0.2950; 0.4686	-	-	-	-	16	0.2976

**Table 2:** The fractions of phases and lattice parameters in alloys after HPT.

Fe, wt.%	*T*, °C	αTi		βTi		ωTi		FeTi	
		*V*, %	*a*, *c*, nm	*V*, %	*a*, nm	*V*, %	*a*, *c*, nm	*V*, %	*a*, nm
0.5	800	11	0.2949, 0.4686	59	0.3259	30	0.4628; 0.2821	-	-
6	580	20	0.2949, 0.4686	31	0.3210	49	0.4624; 0.2816	-	-
6	670	19	0.2949, 0.4686	34	0.3240	47	0.4628; 0.2816	-	-
6	850	14	0.2949, 0.4687	45	0.3239	41	0.4620; 0.2810	-	-
9	470	12	0.2951, 0.4683	18	0.3249	58	0.4631; 0.2821	12	0.2979

**Table 3:** Interpretation of the electron diffraction patterns shown in Figure 6.

Ti–0.5 wt.% Fe	Ti–6 wt.% Fe	Ti–9 wt.% Fe
α (100)	ω (001)	TiFe (100)
ω (101)	ω (101)	α (002)
TiFe_2_ (104)	β (200)	TiFe_2_ (200)
β (200)	TiFe_2_ (204)	TiFe_2_ (202)
TiFe_2_ (211)	α (112)	β (200)
β (211)	TiFe_2_ (220)	α (110)

## Data Availability

The data presented in this study are available upon request from the corresponding author.
